# Kicking KRAS to tackle lung cancer

**DOI:** 10.1038/s43856-021-00017-z

**Published:** 2021-07-23

**Authors:** Ben Abbott

**Affiliations:** Communications Medicine, https://www.nature.com/commsmed

## Abstract

*KRAS* is one of the most commonly mutated oncogenes in lung cancer but has long been considered undruggable. With the recent FDA approval of sotorasib, supported by positive phase II trial data now published in *The New England Journal of Medicine*, this is no longer the case.

**Figure:**
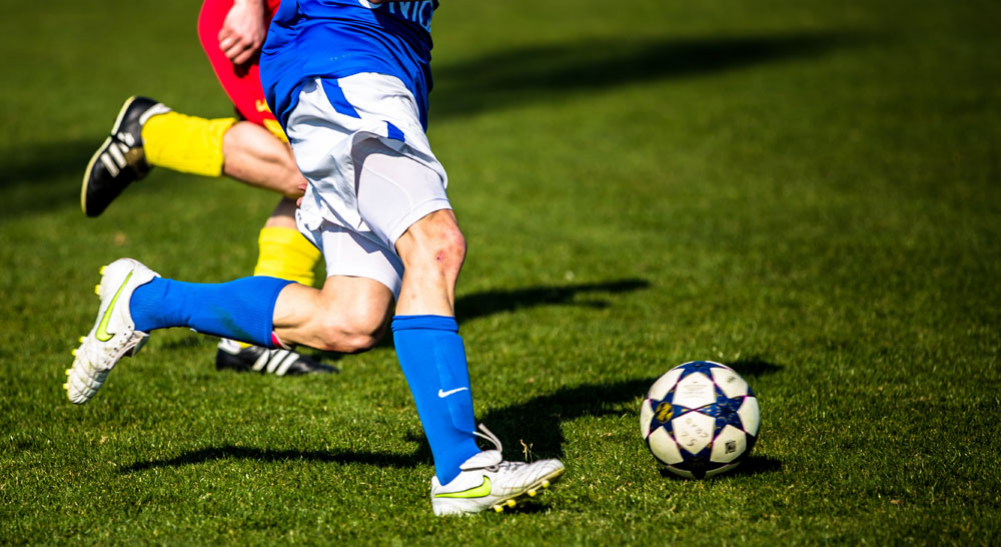
Pixabay

Non-small cell lung cancer (NSCLC) is the leading cause of cancer deaths. Recent advances in targeted therapy and immunotherapy have improved outcomes in subsets of patients, but drugs to target the commonly mutated oncogene *KRAS* have been lacking.

Mutations in *KRAS* occur in ~25% of patients with NSCLC. Around half of these *KRAS*-mutated tumours harbour G12C point mutations, which favour the active GTP-bound state of KRAS to drive downstream oncogenic signalling. *KRAS*^*G12C*^ portends a poor prognosis in individuals with NSCLC and efforts to target the mutant have so far failed, owing to its high affinity for GTP and a lack of binding pockets on the GTP-bound protein.

The phase I portion of the CodeBreaK100 trial previously reported promising safety and efficacy data for sotorasib—a selective and irreversible inhibitor of KRAS^G12C^—with the strongest anti-tumour responses observed in NSCLC patients. Now, Skoulidis and colleagues report findings from the phase II portion of the trial^1^.

In this multicentre, single-arm, open label study, 126 patients with *KRAS*^*G12C*^-mutated NSCLC, who had received prior immunotherapy and/or chemotherapy, were treated with oral sotorasib until disease progression, the onset of unacceptable side effects, or withdrawal of consent. The primary endpoint of objective response rate—a complete or partial reduction in tumour size on imaging, according to RECIST criteria—was evaluable in 124 patients.

At a median follow-up of 15.3 months, the objective response rate was 37.1%. Most responders had a partial response (42/46 patients) and 4 patients had a complete response. Disease control was achieved in 80.6% of all patients and tumour shrinkage of any magnitude occurred in 82.3%. Median progression-free survival was 6.8 months and median overall survival was 12.5 months. Treatment-related adverse events occurred in 69.8% of patients, leading to a dose modification in 22.2% and discontinuation of therapy in 7.1%.

The CodeBreaK100 trial has demonstrated the safety and efficacy of sotorasib in *KRAS*^*G12C*^-mutant NSCLC. Data from the trial supported the FDA’s accelerated approval of sotorasib in May, for patients with *KRAS G12C*-mutated locally advanced or metastatic NSCLC who have received at least one prior systemic therapy. Randomised phase III trials to compare sotorasib with existing therapies and studies to determine potential drug combinations are underway. However, the approval of sotorasib for NSCLC represents a major milestone in cancer therapy and the quest to target this particularly tricky oncogene.

## References

[1] Skoulidis F (2021). Sotorasib for lung cancers with KRAS p.G12C mutation. N. Engl. J. Med..

